# Need Satisfaction and Depressive Symptoms Among University Students in Hong Kong During the COVID-19 Pandemic: Moderating Effects of Positive Youth Development Attributes

**DOI:** 10.3389/fpsyt.2022.931404

**Published:** 2022-07-07

**Authors:** Daniel T. L. Shek, Diya Dou, Xiaoqin Zhu, Tingyin Wong, Lindan Tan

**Affiliations:** Department of Applied Social Sciences, The Hong Kong Polytechnic University, Hong Kong, Hong Kong SAR, China

**Keywords:** depression, positive youth development, beliefs about adversity, psychosocial competence, family functioning

## Abstract

As studies on the mental health status of university students during the COVID-19 pandemic are almost non-existent in Hong Kong, we examined four research questions in this paper: What is the prevalence of depressive symptoms in university students in Hong Kong? What are the socio-demographic correlates of depressive symptoms? Do need satisfaction and positive youth development (PYD) attributes, including beliefs about adversity, psychosocial competence (resilience and emotional competence) and family functioning predict depression? Do PYD attributes moderate the predictive effect of need satisfaction on depression? We examined the above research questions using the Centre for Epidemiologic Studies Depression Scale Revised (CESD-R) in 1,648 university students in Hong Kong. For PYD attributes, we utilized validated measures of Chinese beliefs about adversity, psychosocial competence (resilience and emotional competence), and family functioning. For need satisfaction, we used a measure derived from two focus group interviews involving university students. Results showed that 48.4% of the respondents (95% confidence interval = [45.9%, 51.1%]) scored 16 or above (i.e., “at-risk” for clinical depression). As predicted, age, gender, student status (local vs. international), and family financial hardship were significant socio-demographic correlates of depressive symptoms. Besides, need satisfaction and PYD attributes negatively predicted depression scores. Finally, multiple regression analyses controlling for gender, age, and student status as covariates showed that all PYD attributes moderated the impact of need satisfaction on depression. The findings reinforce the theoretical proposition that PYD attributes serve as important factors in protecting the mental health of university students during the pandemic.

## Introduction

The mental health of university students is a growing concern in the global context (1). In particular, researchers have reported that depression is a common problem in university students. For example, Vázquez and Blanco (2) revealed that 33% of Spanish university students were at-risk of moderate and severe depression. In Ghana, Oppong Asante and Andoh-Arthur (3) found that 31.1% of university students showed mild to moderate depression and 8.1% showed severe depression. Similarly, Tao et al. (4) found that 6.1% of Chinese university students reported at least moderate depressive symptoms. In a prospective longitudinal study conducted in China, Song et al. (5) found that 42% of non-depressed Chinese first-year students developed depressive symptoms within 2 years of college study.

COVID-19 has adversely affected the mental health of students because the learning mode has substantially changed under the pandemic (6). Interestingly, while many researchers have conducted studies in different parts of the world to understand the mental health of university students under the pandemic, related studies are almost non-existent in the Hong Kong context. As the mental health of university students might be adversely affected during the “Social Event” taking place in 2019–2020 (7), there is an urgent need to understand the mental health of Hong Kong university students under the pandemic. In this study, we examined several issues based on a large sample of university students in Hong Kong. These issues included the prevalence and socio-demographic correlates of depressive symptoms in university students, predictive effects of need satisfaction, positive youth development (PYD) attributes and family functioning on depression in college students, and the moderating effect of PYD attributes on the influence of need satisfaction on depression.

## Literature Review

### Prevalence of Depression and Socio-Demographic Correlates of Depression Under COVID-19

According to Shek et al. (8), the COVID-19 pandemic has brought many challenges to university students, such as intrapersonal, interpersonal, academic, and financial adjustments that may lead to increased stress. Obviously, these adjustments within a short time would eventually impair the mental health of university students under the pandemic. There are studies suggesting mental health problems of university students under the pandemic are alarming. Yu et al. (9) showed that 56.8% of Chinese students had moderate or severe levels of depression; Luo et al. (10) showed that 26.0% of Chinese students were at-risk of depression; Truchot et al. (11) showed that 52% of the French female students and 49% of French male students displayed depressive symptoms.

Regarding socio-demographic correlates of depression in university students under COVID-19, researchers have identified several significant correlates. The first correlate is age. Theoretically, with more life experience and better coping abilities (12) as well as resilience (13), older students’ mental health might be better than that of younger students. While many studies supported this hypothesis (14, 15), some studies showed the reverse pattern (16). The second significant demographic correlate is gender. While female college students showed poorer mental health than did male college students under the pandemic (17–19), male students showed more psychological symptoms than did female students (20). A recent meta-analysis also showed mixed findings (1).

The third factor is whether the student comes from abroad. Research generally showed that international students experienced many mental health problems during the pandemic (21) and their mental health was poorer than domestic students (22). Nevertheless, the picture may be different in Hong Kong. Cheung et al. (23) showed that some local students displayed high levels of depression while international students had the lowest level of depression. Furthermore, as the experience of social unrest preceding the pandemic may have already resulted in a deterioration in university students’ mental health (7, 20), the COVID-19 pandemic might further impair the mental health of university students (20, 24).

Finally, college students’ financial distress was significantly associated with anxiety, stress, depression, or post-traumatic stress symptoms [e.g., (25)]. As the pandemic creates a visible financial hardship for people, findings from different places (26, 27) showed that university students experiencing economic hardship had poorer mental health as compared to those without economic disadvantage.

### Need Satisfaction and Positive Youth Development Attributes as Predictors of Depression

Self-determination theory maintains that satisfaction of one’s needs in different aspects (e.g., personal development and social interactions) leads to better adjustment such that need satisfaction results in better mental health while need frustration leads to more mental health problems such as depression (28). There are studies showing need satisfaction was positively related to student mental health (29–31). During the pandemic, university students’ need satisfaction may be threatened due to the interruption of a “normal life” resulting from social distancing measures and other sudden changes in life mode, which may in turn negatively affect their mental health. Hence, besides satisfaction with “basic psychological needs,” we should examine how satisfaction of needs in different life domains would be related to the mental health of university students under the pandemic.

Besides need satisfaction, there are other psychosocial predictors of student mental health under the pandemic. According to the positive youth development (PYD) framework (32), developmental assets such as good family support (33) and attributes including resilience, emotional competence, and optimism (34) are commonly regarded as protective factors of mental health (32). In the present study, we focused on three PYD attributes, including beliefs about adversity, psychosocial competence (resilience and emotional competence), and family functioning. These attributes represent students’ internal as well as external assets that may help them to cope with stressful situations such as the pandemic.

The first PYD attribute is “belief about adversity” that reflects one’s spiritual and positive beliefs about the future when experiencing hardship. Theoretically, holding positive meaning about adversity and positive life orientation can help one cope with stress arising from adversity, including the COVID-19 pandemic (9, 35). The second PYD attribute is “psychosocial competence,” which is operationally defined by resilience and emotional competence in the present study. For resilience, it has been found to be negatively associated with university students’ depression and anxiety during the COVID-19 pandemic (36). Emotional competence also showed a protective role in the context of the COVID-19 pandemic (37, 38). Besides “internal assets” indexed by resilience and psychosocial competence, “external asset” defined by family functioning also protects individual family members (39). There are research findings showing that family functioning was positively related to the mental health of young people under the pandemic (40–42).

### Moderating Effect of Positive Youth Development Attributes

Besides the main effects of the above-mentioned PYD attributes on students’ mental health, PYD attributes may also buffer the negative influence of risk factors (43), including the lack of need satisfaction considered in the present study. There are studies showing significant moderating effect of PYD attributes on the association between risk factors (e.g., stress) and depression (40, 44). Some studies also showed that positive beliefs, such as optimism and meaning-centered coping (e.g., maintenance of hope), mitigated the impacts of COVID-related stress on individuals’ depressive symptoms (45, 46). Nevertheless, there are also studies showing insignificant findings (47, 48) or mixed findings on the moderating effect of PYD attributes on adolescent mental health (49). The inconclusive findings thus call for further exploration of this important issue.

### The Present Study

We asked the following research questions in this study:

Research Question 1: What is the prevalence of depression amongst university students in Hong Kong?

Research Question 2: What are the socio-demographic correlates of depressive symptoms in university students?

• Based on the previous studies that older students showed better coping and resilience than did younger students, we proposed that there would be a negative relationship between age and the level of depressive symptoms (Hypothesis 1a).
• Regarding gender as a correlate, as the findings are inconclusive, we put forward two competing hypotheses: female university students would show a higher level of depression than male university students (Hypothesis 1b-x); male university students would show a higher level of depression than female university students (Hypothesis 1b-y).
• Regarding student status, because there are conflicting findings, we proposed two alternative hypotheses: international students would show a higher level of depression than local students (Hypothesis 1c-x); local students would show more depressive symptoms than international students (Hypothesis 1c-y).
• Based on the existing theoretical frameworks and previous studies, we hypothesized that students experiencing financial difficulty would have a higher level of depression compared to those without such experience (Hypothesis 1d).

Research Question 3: What is the relationship between need satisfaction and depressive symptoms among university students in Hong Kong? With reference to Shek et al. (8) we expected that there would be a negative relationship between these two domains (Hypothesis 2).

Research Question 4: Are PYD attributes related to depressive symptoms? Based on the general thesis that PYD attributes promote youth development, we expected that there would be negative relationships between PYD attributes (positive beliefs about adversity, psychosocial competence, and family functioning) and depressive symptoms (Hypotheses 3a, 3b, and 3c, respectively).

Research Question 5: Do the three PYD attributes moderate the impact of need satisfaction on depression? As PYD attributes are protective factors, we hypothesized that these three PYD attributes would moderate the negative relationship between need satisfaction and depressive symptoms (Hypotheses 4a, 4b, and 4c, respectively).

## Methods

To understand the mental health of university students under the pandemic, we conducted an online survey in the 2020/21 academic year using different measures of mental health (e.g., depression, anxiety, and post-traumatic stress disorder) to examine prevalence rates as well as related socio-demographic correlates. We also examined the risk factors (e.g., stressors in different domains) and protective factors (e.g., positive beliefs about adversity, resilience, emotional management, and family functioning) of student mental health. In this paper, we cover the prevalence of depressive symptoms as well as related socio-demographic correlates in university students in Hong Kong. Besides, we examined the main effect of need satisfaction and PYD attributes and the moderating effect of PYD attributes on the influence of need satisfaction on depressive symptoms.

### Participants and Procedures

We collected data via an online student survey from January 2021 to the end of March 2021, during which the fourth pandemic of COVID-19 in Hong Kong took place. Undergraduate students from one university participated in the study. Although it is desirable to recruit students via random sampling strategies, we were not able to do so in this study for two reasons. First, because of the “work from home” arrangement, it was not easy to get the complete student population list. Second, it was difficult to invite students to join this study via email because their email accounts were flooded with emails during the pandemic. Hence, we recruited participants via quota sampling using faculty and study year to form different categories. Actually, many studies used quota sampling in COVID-19 studies (50–52).

Because of the social distancing requirement, we conducted the online survey via Qualtrics XM. Online surveys have the advantage of flexibility, and they can also motivate participants to disclose information that would not be disclosed under other forms of survey (53, 54). In the online questionnaire, participants first read the information about the study as well as the confidentiality, anonymity, and participants’ rights of the study. If a participant agreed to join after understanding the above information, he/she checked “Yes, I consent to participate in the captioned research.” As an appreciation of their participation, students received a supermarket voucher for successful completion of the survey (HK$100 = roughly US$12.82).

A total of 2,050 students indicated their interest to join the study and 2,017 students met the inclusion criteria (e.g., being an undergraduate student) and gave their consent to join the study. As a measure of quality assurance, we inserted two “attention checking” questions in the questionnaire [e.g., (55, 56)]. In these two questions, we invited the respondents to choose a response option (e.g., “This is an attention check, please choose ‘exactly true”’). Eventually, we excluded 369 cases showing careless responses, with 1,648 students in the final sample.

### Instruments

#### Centre for Epidemiologic Studies Depression Scale Revised

The Centre for Epidemiologic Studies Depression Scale Revised (CESD-R) is a 20-item assessment tool measuring depressive symptoms based on Major Depressive Disorder listed in the DSM-V criteria (57). The original version was developed by Radloff (58). These symptoms include sadness (e.g., “I felt depressed”), anhedonia (e.g., “Nothing made me happy”), appetite problem (e.g., “My appetite was poor”), sleep problem (e.g., “I slept much more than usual”), concentration (e.g., “I could not focus on the important things”), and worthlessness (e.g., “I do not like myself”). There are also items assessing fatigue (e.g., “I was tired all the time”), agitation (e.g., I felt like I was moving too slowly”), and suicidal ideation (e.g., “I wished I were dead”). For each item, respondents were asked to rate their experience in the past week on a five-point scale (“0 = Not at all or less than 1 day in the last week; 1 = 1–2 days in the last week, 2 = 3–4 days in the last week, 3 = 5–7 days in the last week; 4 = nearly every day for the last 2 weeks”). We calculated and interpreted the scale scores according to the instructions on the official website.^1^ There is support for the psychometric properties of the CES-D in the Chinese context (59, 60). The CESD-R was also validated in Chinese samples (61, 62). In the present study, the CESD-R was internally consistent (alpha = 0.96).

#### Need Satisfaction During the Pandemic Scale

To understand the specific needs of university students in Hong Kong, we conducted two focus groups for 22 undergraduate students to facilitate the development of the online questionnaire. Based on the findings, needs in several areas emerged from the findings, including physical needs (e.g., keep physical fitness), psychological needs (e.g., keep good emotional health), social needs (e.g., go out with friends), familial needs (e.g., maintain harmony in family) and academic needs (e.g., have effective online learning strategy). Based on the qualitative data, we developed 15 items. In each item, we asked the respondents how well their needs were met in the past year on a six-point scale (“1 = Not met at all; 6 = Fully met”). Reliability analysis showed that the scale is internally consistent (alpha = 0.89).

#### Chinese Cultural Beliefs About Adversity Scale

We used the Chinese Cultural Beliefs about Adversity Scale designed by Shek et al. (63). There are nine items based on traditional Chinese cultural beliefs (e.g., “hardship increases stature”; “when there is a will, there is a way”). To avoid misunderstanding of the items, in addition to the English version, the original Chinese version was also given for reference. Respondents were required to indicate the degree of agreement on each item by using a 6-point Likert scale (“1 = Strongly disagree, 6 = Strongly agree”). Shek et al. (63) showed that the scale scores were related to measures of psychological well-being. In this study, we found that this scale was reliable (alpha = 0.73).

#### The Chinese Positive Youth Development Scale

We used two subscales in Chinese Positive Youth Development Scale (CPYDS) to assess psychosocial competence, including resilience and emotional competence (64). There are three items in the resilience subscale (e.g., “I would not give up easily even in face of difficulties”) and three items in the emotional competence subscale (e.g., “I know how to ventilate my emotions appropriately in times of distress”). Respondents were required to indicate their level of agreement on the six items on a 6-point Likert scale ranging from “1” (Strongly disagree) to “6” (Strongly agree). The resilience and emotional competence subscales showed acceptable internal consistency (alpha = 0.78 and 0.81, respectively). We computed the mean score of these two subscales to indicate the construct of “psychosocial competence” (alpha = 0.86).

#### The Chinese Family Assessment Instrument

We used the 9-item Chinese Family Assessment Instrument (C-FAI) to assess family functioning in this study (65), including three items on family communication (e.g., “Parents often talk to their children”), three items on mutuality (e.g., “Family members love each other”) and three items on conflict (e.g., “There is no mutual concern among family members”). We asked the respondents to indicate their level of agreement with each statement on a 5-point Likert scale ranging from “1” (Very unlike my family) to “5” (Very like my family). This 9-item measure shows good reliability in this study (alpha = 0.77).

## Results

The mean age of the final sample (*N* = 1,648) was 20.09 years, with 696 (42.23%) male students and 854 (51.82%) female students. The remaining 98 (5.95%) participants did not indicate their gender in the questionnaire. Most of the students (*N* = 1,613; 97.88%) were local students and 35 (2.12%) were international students, mainly from mainland China and Malaysia. Some students (*N* = 351; 21.3%) indicated that their families experienced financial hardship at the time they completed the survey. Tables 1, 2 show the demographic characteristics of the sample and the descriptive statistics of the variables of the study, respectively.

**TABLE 1:** Demographic characteristics of the sample.

	Valid number	%
N (participants)	1,648	
**Gender**		
Male	696	42.23
Female	854	51.82
Missing value	98	5.95
**Age (mean = 20.09 years)**		
Below mean	1,074	65.17
Above mean	573	34.77
Missing value	1	0.06
**Student status**		
Local	1,613	97.88
International	35	2.12
**Family financial difficulty**		
Has financial difficulty	1,100	66.75
Without financial difficulty	351	21.30
Missing value	197	11.95

**TABLE 2:** Statistics of mean, SD, reliability, and correlations.

	Variables	Mean (SD)	Cronbach’s α (mean inter-item correlations)	1	2	3	4	5	6	7	8
1	Age	20.09 (1.37)	–								
2	Gender^a^	–	–	–0.02							
3	Student status^b^	–	–	0.13***	0.06*						
4	Family financial difficulty^c^	–	–	–0.003	0.02	–0.01					
5	Need satisfaction	3.78 (0.73)	0.89 (0.34)	–0.05	0.07*	0.09***	–0.15***				
6	Beliefs about adversity	3.89 (0.65)	0.73 (0.24)	0.03	0.20***	0.14***	–0.08**	0.37***			
7	Psychosocial competence	4.00 (0.78)	0.86 (0.51)	0.06*	0.11***	0.13***	–0.07*	0.50***	0.57***		
8	Family functioning	3.31 (0.58)	0.77 (0.27)	–0.04	0.08**	0.12***	–0.18***	0.36***	0.31***	0.32***	
9	Depression	18.82 (15.21)	0.96 (0.52)	–0.08**	–0.10***	–0.08**	0.25***	–0.26***	–0.34***	–0.38***	–0.22***

*^a^Male = 1, Female = 2.*

*^b^Local student = 1, International student = 2.*

*^c^Do not experience financial difficulty = 0, Experience financial difficulty = 1.*

*p < 0.05; **p < 0.01; ***p < 0.001.

Among the 1,648 participants, 48.4% (95% CI = [45.9%, 51.1%]) scored 16 or higher in CESD-R (i.e., at-risk for clinical depression). Regarding the socio-demographic correlates of depressive symptoms (Table 3), younger participants (*M* = 19.82, *SD* = 15.36) scored higher depression scores than did older participants (*M* = 16.96, *SD* = 14.75; *F* = 13.30, *p* < 0.001, *η^2^*_*p*_ = 0.01), providing support for Hypothesis 1a. For gender differences in depressive symptoms, male students (*M* = 20.79, *SD* = 15.82) displayed a higher level of depressive symptoms than did female students (*M* = 17.76, *SD* = 14.63; *F* = 15.26, *p* < 0.001, *η^2^*_*p*_ = 0.01), lending support to Hypothesis 1b-y. For differences between international and local students, local Hong Kong students (*M* = 19.00, *SD* = 15.25) showed more depressive symptoms than did international students (*M* = 10.71, *SD* = 9.95; *F* = 10.23, *p* < 0.01, *η^2^*_*p*_ = 0.01), giving support to Hypothesis 1c-y. Finally, supporting Hypothesis 1d, students experiencing financial hardship (*M* = 25.20, *SD* = 16.87) showed more depressive symptoms than did students without such an experience (*M* = 16.42, *SD* = 13.60; *F* = 98.15, *p* < 0.001, η^2^_*p*_ = 0.06).

**TABLE 3:** Results of UNIANOVA.

	N	Mean	SD	F	Partial eta squared
**Age group**					
Below mean	1,074	19.82	15.36	13.30***	0.01
Above mean	573	16.96	14.75		
**Gender**					
Male	696	20.79	15.82	15.26***	0.01
Female	854	17.76	14.63		
**Student status**					
Local student	1,613	19.00	15.25	10.23**	0.01
International student	35	10.71	9.95		
**Family economic difficulty**					
Do not have difficulty	1,100	16.42	13.60	98.15***	0.06
Have difficulty	351	25.20	16.87		

**p < 0.01; *** p < 0.001.

As predicted, need satisfaction, beliefs about adversity, psychosocial competence, and family functioning were negatively related to depression (see Table 2). To understand the predictive effect of these factors on depression, hierarchical multiple regression analyses were conducted with age, gender, student status, and financial difficulty as covariates. In Step 1, all covariates were entered as a block. Then we added each predictor separately in Model 2 to Model 5 (see Table 4). Results showed that these factors predicted depression in the expected direction (Hypothesis 2 and Hypotheses 3a to 3c).

**TABLE 4:** The predictive effects of need satisfaction and PYD attributes on depression.

Model	Predictors	β	*t*	Cohen’s *f*^2^	R^2^ change	F change
						
1	Age	–0.08	−3.07**	0.01	0.08	30.70***
	Gender^a^	–0.01	−3.77***	0.01		
	Student status^b^	–0.06	−2.24*	0.004		
	Family financial difficulty^c^	0.25	9.58***	0.07		
2	Need satisfaction	–0.23	−8.70***	0.06	0.05	75.68***
3	Beliefs about adversity	–0.32	−12.82***	0.12	0.10	164.26***
4	Psychosocial competence	–0.37	−14.90***	0.16	0.13	221.91***
5	Family functioning	–0.18	−6.99***	0.04	0.03	48.90***

*In Models 2–5, control variables (age, gender, student status, and family financial difficulty) were statistically controlled.*

*^a^1 = male, 2 = female.*

*^b^1 = Local students, 2 = International students.*

*^c^0 = did not experience economic difficulties, 1 = experienced economic difficulties.*

*p < 0.05; ** p < 0.01; *** p < 0.001.

To examine the main and moderating effects of PYD attributes, we conducted three multiple regression analyses (see Table 5). In each model, we examined the effects of need satisfaction and PYD attributes. Findings provided support for the significant main effects of need satisfaction (β = –0.23, *p* < 0.001, Cohen’s *f*^2^ = 0.06) and PYD attributes (β ranged between –0.34 and –0.23, *p* < 0.001, Cohen’s *f*^2^ ranged between 0.01 and 0.11). Results also showed significant interaction effects of need satisfaction and PYD attributes (β ranged between –0.91 and –0.70, *p* < 0.001, Cohen’s *f*^2^ = 0.01). The findings support Hypotheses 4a to 4c.

**TABLE 5:** The predictive effects of need satisfaction on depression and the moderating effect of three PYD attributes.

Model	Predictors	Beliefs about adversity			Psychosocial competence			Family functioning		
		β	*t*	Cohen’s *f*^2^	β	*t*	Cohen’s *f*^2^	β	*t*	Cohen’s *f*^2^
Model 1	Age	–0.08	−3.07**	0.01	–0.08	−3.07**	0.01	–0.08	−3.07**	0.01
	Gender^a^	–0.10	−3.77***	0.01	–0.10	−3.77***	0.01	–0.10	−3.77***	0.01
	Student status^b^	–0.06	−2.24*	0.003	–0.06	−2.24*	0.003	–0.06	−2.24*	0.003
	Family financial difficulty^c^	0.25	9.58***	0.07	0.25	9.58***	0.07	0.25	9.58***	0.07
	R^2^ change	0.08			0.08			0.08		
	F change	30.70***			30.70***			30.70***		
Model 2	Need satisfaction	–0.23	−8.70***	0.06	–0.23	−8.70***	0.06	–0.23	−8.70***	0.06
	R^2^ change	0.05			0.05			0.05		
	F change	75.68***			75.68***			75.68***		
Model 3	PYD attributes	–0.28	−10.36***	0.08	–0.34	−11.96***	0.11	–0.12	−4.42***	0.01
	R^2^ change	0.06			0.08			0.01		
	F change	107.33***			142.93***			19.52***		
Model 4	Need satisfaction × PYD attributes	–0.89	−4.44***	0.01	–0.70	−4.21***	0.01	–0.91	−4.37***	0.01
	R^2^ change	0.01			0.01			0.01		
	F change	19.67***			17.73***			19.08***		

*In Models 2–4, age, gender, student status, and family financial difficulty were statistically controlled. Based on Model 1, need satisfaction was added to Model 2. Based on Model 2, each PYD attribute was included separately in Model 3. Based on Model 3, the interaction of need satisfaction and the respective PYD attribute was further included in Models 4.*

*p < 0.05; **p < 0.01; ***p < 0.001.

**FIGURE 1:**
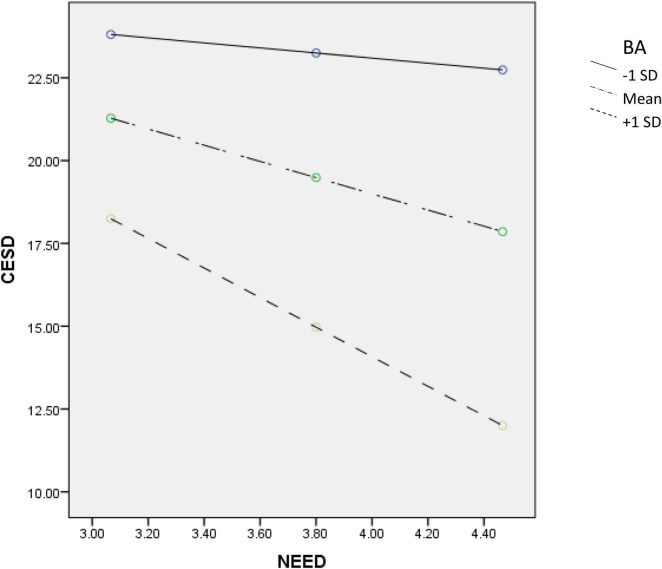
Moderating effect of BA (beliefs about adversity) on the relationship between need satisfaction and depression.

**FIGURE 2:**
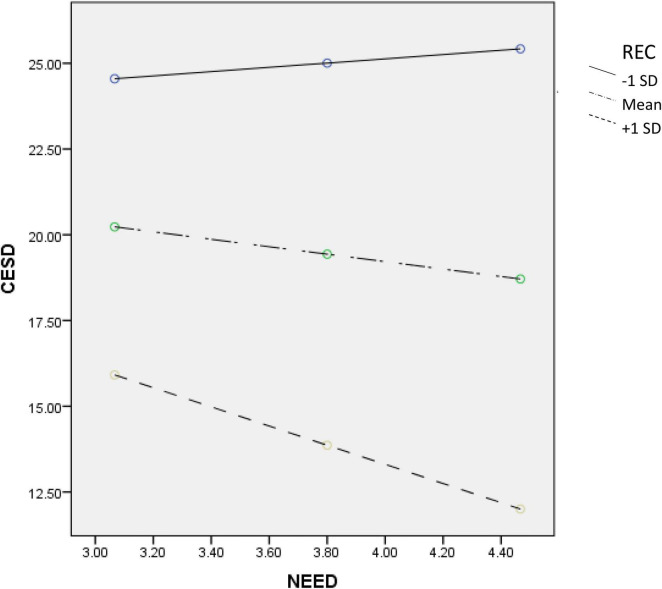
Moderating effect of REC (psychosocial competence) on the relationship between need satisfaction and depression.

**FIGURE 3:**
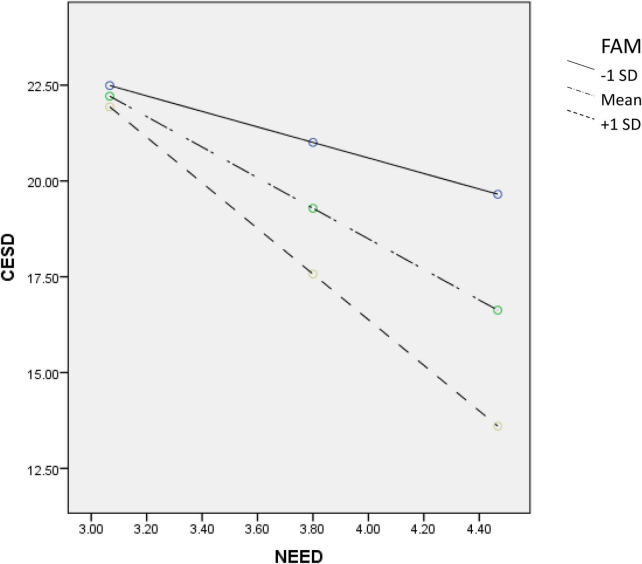
Moderating effect of FAM (family functioning) on the relationship between need satisfaction and depression.

To understand the moderating effect of PYD attributes, we conducted simple slope analyses to check the predictive effect of need satisfaction on depression based on participants with high (scored one standard deviation above the mean score) or low (scored one standard deviation below the mean score) level on each PYD attribute. First, need satisfaction showed a significant negative predictive effect on depression among students with more positive beliefs about adversity (*B* = –4.46, *SE* = 0.69, *p* < 0.001, 95% confidence interval (CI) = [–5.82, –3.11]) but not among students with less positive beliefs about adversity (*B* = –0.76, *SE* = 0.68, *p* = 0.26, 95% CI = [–2.09, 0.56]). Second, need satisfaction only served as a negative predictor of depression among students with high psychosocial competence (*B* = –2.79, *SE* = 0.69, *p* < 0.001, 95% CI = [–4.15, –1.44]) but not among students with low psychological competence (*B* = 0.62, *SE* = 0.71, *p* = 0.38, 95% CI = [–0.78, 2.02]). Finally, need satisfaction demonstrated a stronger negative prediction on depression among students with better family functioning (*B* = –5.95, *SE* = 0.75, *p* < 0.001, 95% CI = [–7.42, –4.49]) than among those with poor family functioning (*B* = –2.03, *SE* = 0.68, *p* < 0.01, 95% CI = [–3.36, –0.69]). Figures 1–3 depict the moderating effect of these three PYD attributes.

## Discussion

With reference to the limitations of the literature, this study has several advances. First, while studies on university students’ mental health have been conducted in mainland China, there are relatively fewer studies in Hong Kong. Second, using the CESD-R, we obtained a picture of depression among university students during the COVID-19 pandemic, which has practical service implications. Third, we identified socio-demographic correlates of depressive symptoms in university students in Hong Kong. Fourth, instead of examining basic psychological need satisfaction, we examined need satisfaction in different life domains during the pandemic. Fifth, we recruited a large sample of students for this study. Sixth, we examined the relationships between PYD attributes and depression in university students, which are not systematically examined in the literature. Finally, the present findings highlighted the moderating role of PYD attributes on the impact of need satisfaction on depressive symptoms.

Regarding Research Question 1, the finding that 48.4% of the respondents were at-risk for clinical depression deserves attention. While the prevalence rate in the present study was similar to those reported in some Western studies during the pandemic [e.g., (66)], it is also lower or higher than the prevalence rates reported previously based on Chinese university students (9, 10). Of course, we should be cautious in interpreting the prevalence rates using the CES-D across different studies because different versions of CES-D, samples, languages, and timing during the pandemic were involved. Nevertheless, the present findings suggest that depression is a common risk amongst university students in Hong Kong during the pandemic that calls for intervention.

For Research Question 2, there are several interesting observations. Consistent with previous findings (67), younger students showed a higher level of depression than old students. This observation can possibly be explained in terms of higher coping abilities amongst the older students (12). Furthermore, young students may not be familiar with the university environment and related facilities, which would contribute to their relatively higher depression level. For gender differences in depression, in contrast to the common finding that female college students showed poorer mental health, we found that male students displayed more depressive symptoms than did female students. Several factors may contribute to this finding (68, 69). First, as Hong Kong is an achievement-oriented society, males experience greater stress than females because males are expected to be successful in the Chinese culture. Second, Chinese men used to suppress their negative emotions. Third, Chinese men also tended not to seek help when encountering problems. Actually, previous studies also showed that males showed poorer mental health as compared to female students (20). Concerning student status, in contrast to the common belief that international students would experience more stress than local students, we found that Hong Kong students showed more depressive symptoms than did international students. There are three possible factors contributing to this observation. First, the mental health problems of university students in Hong Kong have been alarming historically (20). Second, because of social unrest and COVID-19, Hong Kong university students actually experience “cumulative stresses.” Third, Hong Kong people are facing many stressors under COVID-19, such as having stressful online classes because of the tight living space in Hong Kong (8).

Consistent with the literature, financial difficulty is a risk factor for depression among university students. Theoretically, family economic burdens would adversely affect family processes (e.g., family functioning and parenting) which would eventually impair the mental health of children (70). Under COVID-19, because of city lockdown and social distancing measures, the economy is adversely affected. In Hong Kong, financial difficulty is particularly intense for three reasons. First, there is no unemployment benefit in Hong Kong. Hence, unemployment under COVID-19 is a big problem for families in Hong Kong, particularly grass-root families. Although the Government has launched employment protection schemes, only those who are employed are protected. Second, as university students commonly take up part-time jobs and such job opportunities drop under COVID-19, reduced income is a problem for university students. Third, as the cost of living in Hong Kong is very high, financial strain under COVID-19 is particularly painful.

With regard to Research Question 3, we found that need satisfaction was negatively related to depressive symptoms as predicted. As we have pointed out, while there are some studies examining the relationships between “general” or “basic” psychological needs and mental health, we found that satisfaction of “specific” needs was related to mental health in the present study. As the inability to satisfy needs can be regarded as “daily hassles,” the present findings are consistent with the literature that daily hassles were positively related to mental health problems (71, 72). Theoretically, the present findings are in line with the theoretical proposition that satisfaction of basic psychological needs contributes to mental health (73). However, the present findings go beyond to show that need satisfaction with real-life challenges under COVID-19 is related to depression. Practically, university administrators and teachers have to figure out ways to meet the practical needs of the students.

Concerning Research Question 4, we found that PYD attributes indexed by the three measures were negatively related to depressive symptoms. Generally speaking, the findings are in line with the PYD literature demonstrating the protective effects of PYD attributes, including belief about adversity, emotional competence, and family functioning, on adolescents’ overall development (32) and mental health under the COVID-19 (41, 74). These findings are also consistent with the theoretical proposition that developmental assets such as PYD attributes promote holistic youth development such as positive mental health (75).

The first PYD attribute covered in this study is beliefs about adversity. The present finding provides support for the theoretical propositions that hope (76) or life meaning (77) are important factors helping people to adjust to life adversities. A special feature of this study is that we use indigenous Chinese beliefs about adversity that could strengthen the ecological validity of the measure. Obviously, how to remain hopeful and find out meaning in life under COVID-19 is important (78). In particular, the present findings echo the argument that cultural resources could help people to deal with adversity (79, 80).

We covered psychosocial competence as the second PYD attribute in this study. Theoretically, the importance of competence is highlighted in different PYD models (32). Empirically, there are studies showing that psychosocial competence contributes to positive development in young people. For example, in a meta-analysis, Durlak et al. (81) reported that compared to control participants, students joining social-emotional learning programs showed better developmental outcomes. With reference to Hong Kong, studies based on high school students showed that curricular-based PYD programs could promote the development of adolescents (82). In the university context, research findings also showed that credit-bearing subjects utilizing PYD principles were effective in promoting psychosocial competence in students (83, 84). Obviously, as there is much support for the effectiveness of social-emotional learning programs (85), there is a need to “inoculate” university students against the harmful effects of adversity by developing and implementing PYD programs.

Finally, in line with family functioning theories (86), family functioning was negatively associated with depressive symptoms. However, although family functioning is an important factor shaping adolescent development, systematic research work is not rich, particularly in the Chinese context. In fact, one can argue that although COIVD-19 may lead to a drop in “family financial capital,” healthy family functioning can promote “family social capital” that can lead to the healthy development of young people. With particular reference to the Chinese culture, families are strongly emphasized as the core socialization base for children in a family. Nevertheless, there are three common problems within Chinese families. First, family communication is not open as Chinese people believe that “taboo” topics (e.g., parental problems) should not be openly discussed. Second, as Chinese culture emphasizes interpersonal harmony, expression of negative emotions is not commonly encouraged. Third, there are inter-generational differences in viewing family responsibilities. Obviously, it is important to promote family functioning in university students. However, as university students are commonly regarded as “grown-up” individuals, there are very few related programs for them.

As for research question 5, the findings revealed significant moderating effects of PYD attributes in shaping the relationship between students’ need satisfaction and their depression. For students with higher levels of PYD attributes, the negative relationship between need satisfaction and depressive symptoms was stronger than that for those with lower levels of PYD attributes. These findings echo the general theoretical prediction of PYD models that both internal assets (such as emotional competence, resilience, and beliefs about adversity) and external developmental assets (such as family functioning) protect adolescent mental health (32). Overall speaking, the present finding provides support that the theoretical view that a higher level of developmental assets is a protective factor for adolescent development [(87), p. 894].

The finding based on Chinese cultural belief about adversity broadens our understanding of the theoretical mechanisms involved in moderation. Of course, how to promote Chinese beliefs about adversity in Chinese young people when they do not endorse the Chinese culture (7) is an important point for reflection. For psychosocial competence, the present findings reinforce the notion that PYD attributes can be regarded as the “royal road” to optimal development in young people. In addition to resilience and emotional competence covered in this study, it would be exciting to understand how other PYD attributes, such as positive identity and cognitive competence, would contribute to thriving. Finally, the present study enriches the family ecological approach that family functioning is a key in promoting the wellbeing of young people. Obviously, good family functioning constitutes life meaning (e.g., maintaining a happy family and loving each other) and provides hope for an individual. Hence, how to promote family functioning is important during the COVID-19 pandemic, particularly when family members have to spend more time at home because of social distancing measures.

Despite the pioneering nature and the theoretical as well as practical implications of the study, there are several limitations of the study. First, although self-report measures are widely used, it has been criticized for low validity (88). However, Chan (89) argued that these limitations may have been exaggerated, claiming that “these errors may also apply to non-self-report measures” (p. 330), implying the importance of critically assessing the weaknesses (or strengths) of self-report data. Second, the present study is a cross-sectional study. However, cross-sectional studies are useful because they are relatively inexpensive, less time-consuming, and easy to perform (90, 91). Employing a cross-sectional design in this study can provide a “snapshot” of outcomes and features associated with COVID-19 related depressive symptoms and other mental health risk factors among university students at a particular point in time (90). Third, quota sampling instead of stratified random sampling was used in this study. Again, many studies, particularly during the COVID-19 pandemic, use quota sampling approach, with some researchers believing that this sampling method can generate representative samples. As commented by Sharma (92), using quota sampling has certain limitations (e.g., lack of random selection), but it is particularly useful when researchers are not able to get a probability sample but still want to generate a sample that’s “mirrors” the population being researched. Despite these limitations, this study is a pioneering attempt to understand depression in university students and its related socio-demographic correlates, need satisfaction, and PYD attributes in university students in Hong Kong.

## Data Availability Statement

The raw data supporting the conclusions of this article will be made available by the authors, without undue reservation.

## Ethics Statement

The studies involving human participants were reviewed and approved by the Institutional Review Board (or its Delegate) at the Hong Kong Polytechnic University. The patients/participants provided their written informed consent to participate in this study.

## Author Contributions

DS contributed to all steps of the work. DD and XZ contributed to the project implementation, data interpretation, and revising the work. TW and LT helped draft part of the work. All authors approved the final version of the manuscript.

## Conflict of Interest

The authors declare that the research was conducted in the absence of any commercial or financial relationships that could be construed as a potential conflict of interest.

## Publisher’s Note

All claims expressed in this article are solely those of the authors and do not necessarily represent those of their affiliated organizations, or those of the publisher, the editors and the reviewers. Any product that may be evaluated in this article, or claim that may be made by its manufacturer, is not guaranteed or endorsed by the publisher.

## Footnotes

1
http://cesd-r.com
